# Difficult to treat purulent pericarditis – where does full sternotomy stand? A case report

**DOI:** 10.1097/RC9.0000000000000005

**Published:** 2026-01-08

**Authors:** João Aquino, Inês Alves, João Roque

**Affiliations:** Department of cardiothoracic surgery, Santa Cruz Hospital, Western Lisbon Local Health Unit, Lisbon, Portugal. Portugal

**Keywords:** cardiac surgery, case report, purulent pericarditis, pyopericardium, sternotomy

## Abstract

**Introduction:**

Purulent pericarditis is a subtype of acute pericarditis with a high mortality even when adequately treated. Standard therapy is a combination of antibiotics and drainage, which can be more or less invasive. This is a case report of a patient with purulent pericarditis where cardiac surgery via sternotomy for thorough debridement and lavage was required for complete infection control

**Case presentation:**

A 41-year-old man with a history of injected and inhaled drug use was admitted with a large pericardial effusion, and 700 mL of pus was percutaneously drained. After being started on broad spectrum antibiotics the patient remained with persistent signs of infection, and was submitted to surgical drainage and debridement via full median sternotomy. The patient improved progressively afterwards, and after agent identification and being started on intravenous penicillin, he was discharged after 4 weeks, without evidence of wound infection

**Clinical Discussion:**

An organized pleural effusion was likely the cause of the purulent pericarditis. Of all invasive treatment options available, full median sternotomy was considered the most appropriate for this patient, as it allowed for maximal exposure for debridement. Even though the patient was operated in an actively infected state, there was no wound infection or dehiscence, in part because of meticulous perioperative wound care

**Conclusion:**

Purulent pericarditis is a fatal disease, and cardiac surgery via full median sternotomy may be necessary and at times the most appropriate for adequate complete treatment, as it is simple, easily reproducible, and with an acceptable risk of wound infection

## Introduction

Purulent pericarditis, or pyopericardium, is a very rare subtype of acute pericarditis, accounting for less than 1% of cases^[1]^, and is to this day a severe disease with high mortality even with adequate therapy^[2]^. Treatment includes a combination of antibiotics and drainage, which can be more or less invasive, traditionally requiring cardiac surgery to some extent mainly in refractory or relapsing cases^[3]^. The level of invasiveness remains a point of contention, as shown by the low level of evidence in current guidelines^[3]^, and some groups advocate for simple percutaneous drainage^[4]^, some recommend drainage combined with lytic enzyme instillation^[5,6]^, others subxyphoid^[7]^ or thoracoscopic percardiotomy or pericardial excision^[8]^. Concerns remain about the morbidity of full sternotomy and complete pericardiectomy in the treatment of purulent pericarditis, although the latter is definitely recommended when constrictive pericarditis has occurred^[3]^.

**HIGHLIGHTS**

- Purulent pericarditis is a rare disease with high mortality, and standard treatment consists of pericardial drainage and antibiotics.
- This is a case report of a 41-year-old immunocompetent male patient who developed purulent pericardial effusion that failed to improve completely after standard treatment.
- Complete clinical improvement was only achieved after undergoing full median sternotomy for drainage and thorough debridement followed by a directed and prolonged course of antibiotics.
- Of all invasive treatment options available, full median sternotomy allows for maximal pericardial exposure and the most thorough debridement and drainage possible.
- Median sternotomy is simple and readily available with little equipment necessary.

This is a case report of a high-risk patient with purulent pericarditis where cardiac surgery via full median sternotomy for thorough debridement and lavage was required for complete and sustained infection control, highlighting the potential role of aggressive surgical intervention when standard drainage may be insufficient, well before the onset of constrictive pericarditis.

This case report has been reported in line with the SCARE checklist^[9]^.

## Case presentation

A 41-year-old man with a known history of intravenous heroin and inhaled cocaine use, heavy tobacco use and recent homelessness, no allergies and no known medication or other relevant past history presented to the local emergency department with symptoms of constant piercing thoracic pain and persistent fever in the last 5 days. On physical examination, the patient was restless, diaphoretic, with hypophonic heart sounds on auscultation. He was tachycardic (heart rate of 110 bpm) and with fever (38°C axillary temperature). The blood pressure was 126/78 mmHg. Arterial blood gases were normal, with normal lactate (2.47 mmol/L) and 95% oxygen saturation with no supplementation. The electrocardiogram showed diffuse ST segment elevation. A point-of-care cardiac ultrasound revealed a large pericardial effusion with some signs of hemodynamic compromise.

**Figure 1.**
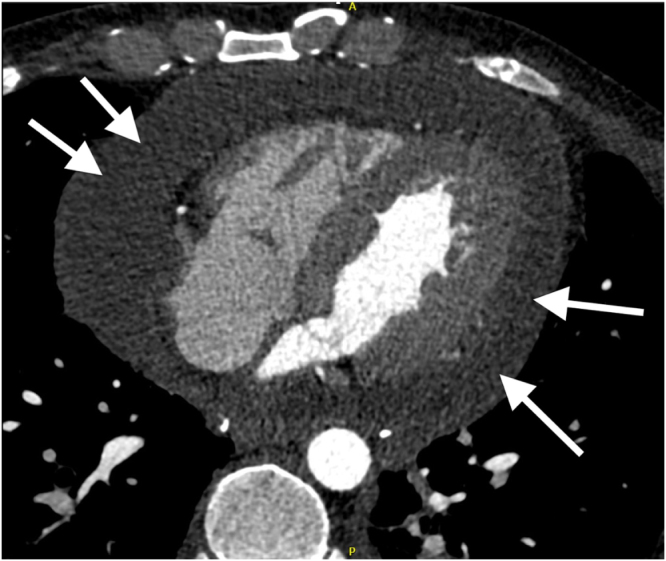
Computerized tomography scan showing extensive pericardial effusion (white arrows).

The patient was then transferred to a tertiary cardiac intensive care unit, further exams were carried out as follows:

Cardiac computerized tomography scan ruled out coronary disease, and showed a significant pericardial effusion and organized right pleural effusion (Fig. 1);

Complete blood count revealed leukocytosis (33 × 10^9^/L); and biochemistry elevated ferritin (1900 mg/dL); C reactive protein (19.8 mg/dL); procalcitonin (6.43 ng/mL) and acute kidney injury, with elevated creatinine (1.77 mg/dL).

All common serologies were negative, namely HIV-1 or HIV-2.

The patient was started on ibuprofen 600 mg three times daily and colchicine 0.5 mg twice daily; however, by day 3 of admission, he remained diaphoretic, tachycardic, and by then with periods of atrial fibrillation.

A diagnostic and therapeutic bedside percutaneous pericardiocentesis was performed. And 700 mL of frank pus were extracted, and sent for culture. The patient was started on piperacillin/tazobactam 4500 mg four times daily and vancomycin 1100 mg three times daily and adjusted to blood levels.

**Figure 2.**
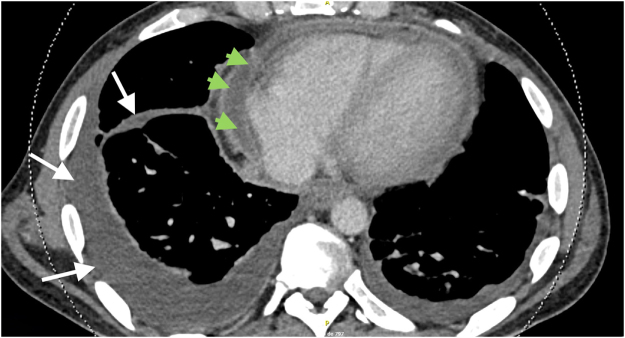
Computerized tomography scan showing organized pleural effusion (white arrows) and moderate pericardial effusion (green arrowheads).

Over the following 2 days, the complete blood count and biochemistry showed persistent leukocytosis (maximum 44 × 10^9^/L) and persistently elevated c reactive protein (23.4–21.3 mg/dL) and procalcitonin (17.5 ng/mL). A thoracic computed tomography scan revealed moderate but organized right pleural effusion, and residual pericardial effusion (Fig. 2). Since the patient had no respiratory symptoms and the pleural effusion was moderate and likely loculated, no attempt of pleural drainage was attempted.

Cardiac surgery was consulted for a more thorough surgical pericardial drainage, and the patient was admitted to the operating room on day 6 of admission.

**Figure 3.**
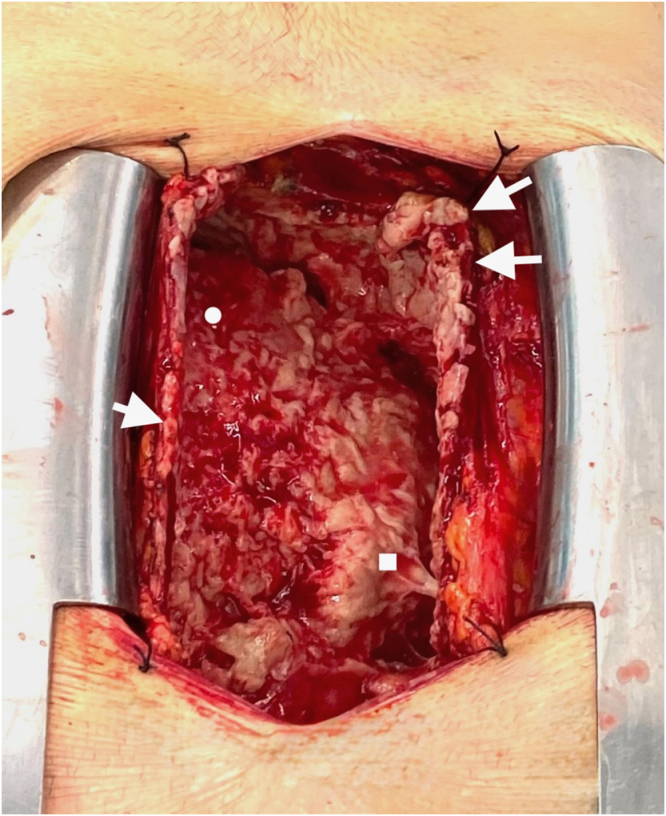
Intraoperative view of pericardium (white arrows) right atrium (white square) and right ventricle (white circle). The pericardium end epicardium are both quite thick and covered in dense exudative plaques.

The patient was positioned in dorsal decubitus and prepped and draped. A full median sternotomy was performed. The mediastinal tissues were oedematous, and the fibrous pericardium was tense. A small opening was created, and approximately 500 mL of pus under tension was immediately drained. The pericardium was then fully opened. The parietal pericardium was thickened and the epicardium was covered in thick exudate and white plaques (Fig. 3). Using blunt and sharp dissection and ample quantities of warm saline the surgical team removed as much infected tissue as possible without injury to the heart, and partially resected the anterior parietal pericardium. Both pleural spaces appeared to have chronic adhesions and were not opened. Two large bore drains were left in the pericardium and mediastinum, the fibrous pericardium was loosely approximated with three simple polydioxanone sutures and the sternum was closed with six overlapping figure-of-eight sternal wires.

In the cardiac surgery intensive care unit, the patient was maintained on broad spectrum antibiotics, and needed noradrenaline briefly for pressure control, but was extubated 4 h after admission. The postoperative blood count showed an improvement in leukocytosis (23 × 10^9^/L); and biochemistry showed a progressive normalization of c reactive protein (16.7 mg/dL) and procalcitonin (4.5 ng/mL). The patient was transferred to the cardiac surgery ward in the following day, and the drains were removed 7 days after the procedure, once the drainage was minimal.

Crucially, the pus cultures revealed *Streptococcus pneumoniae* sensitive to penicillin (minimal inhibitory concentration 0.0016 mg/L), and the patient was started on intravenous benzylpenicillin at a dose of 12 MU/day for 4 weeks.

The remainder of the patient’s admission was unremarkable, with no complications, and he was successfully discharged with oral colchicine and ibuprofen after completing 4 weeks of intravenous benzylpenicillin. There was no sternal wound infection or dehiscence during this period, and wound healing has been favourable up to 3 months after the procedure.

The timeline summarizing the relevant events of this case is represented in Table 1.

**Table 1:** Timeline of events

Day 0		Day 1	Day 3	Day 6		Day 7
Admission to emergency department	Admission to cardiology intensive care unit	Oral ibuprofen and colchicine	Pericardiocentesis. Patient is started on broad spectrum antibiotics	Cardiacsurgery	Extubation. Wean from vasopressors.	Discharge from cardiac surgery intensive care unit
**Day 8**	**Day 13**	**Day 15**	**Day 36**			
Patient started on benzylpenicillin 12 MU/day	Drain removal	Patient transferred to general ward	Patient discharged from hospital.			

## Discussion

Purulent pericarditis usually develops from locally invasive valve endocarditis^[10]^; local dissemination from pneumonia, empyema^[11],^ or liver abscesses^[12]^; hematogenous spread^[13]^; or other rarer causes^[14]^. In this patient’s case, an organized right pleural effusion may have been the source of the pericardial effusion, despite showing no clinical signs or symptoms of active lung or pleural infection.

As the patient still had clinical and radiologic evidence of active infection after standard therapy with percutaneous drainage and broad-spectrum antibiotics, a more invasive and definite treatment was definitely required, and cardiac surgery was promptly consulted. This is in accordance with the most recent European guidelines for pericardial disease^[3]^.

A full median sternotomy with ample pericardial opening revealed a severely infected and thickened pericardium and epicardium, and a thorough debridement and large bore drainage were required to adequately control the infection. The patient only improved fully after this intervention. It is of importance to emphasize that even though the patient was operated in a severely infected state, there were no deep or superficial sternal wound infections or wound dehiscence.

The rarity of this disease precludes large databases and there are very few clinical trials comparing therapeutic options. Because of this, management has been based on case reports and small case series, such as the work from Megged *et al*^[4]^, Dybowska *et al*^[6]^; Sagristà-Sauleda *et al*^[7]^ and Liem *et al*^[8]^. Cui *et al* conducted one very small clinical trial comparing standard invasive treatment to percutaneous treatment with fibrinolytic administration^[15]^. While the results of fibrinolytic administration were satisfactory, they were not superior to invasive treatment, and are not generalizable, since only 34 patients with purulent pericarditis were recruited.

Considering the various surgical options available, our group chose the one that was the simplest in an emergency setting while also taking into consideration that a subxiphoid approach would not allow for enough exposure, and thoracoscopic or open pleural-pericardial windows would add unnecessary risk of lung injury, since there was radiologic evidence of organized pleural adhesions, which were confirmed intraoperatively.

It is of importance to address the issue of sternal wound infection in such cases. Purulent pericarditis specific risk estimation of sternal wound infection is necessarily based on small case reports and case series. Therefore, for this patient, postoperative sternal wound infection risk was managed by strict adherence to relevant guidelines by Lazar et al. and endorsed by the American Association of Thoracic Surgeons^[16]^, and included smoking cessation during hospitalization, preoperative chlorohexidine bath, antibiotic treatment and prophylaxis, avoidance of bone wax, figure-of-eight wire sutures for sternal closure, strict perioperative glycaemic control, among others. Finally, this work has important limitations. It is a case report, and therefore this successful case is not generalizable, and long-term follow up of this patient is lacking.

## Conclusion

Purulent pericarditis is a rare and often fatal disease, and when the usual treatment of percutaneous drainage and large spectrum antibiotics is proven insufficient, prompt cardiac surgery via full median sternotomy may not only be necessary, but in certain situations advisable instead of other approaches, as it allows for maximum exposure for thorough debridement whilst being simple, easily reproducible and with an acceptable risk of wound infection.

Treatment of this disease in a tertiary hospital with the necessary specialties and timely access to them is warranted.

## Data Availability

The protocol and data analyzed in this study are available after adequate anonimysation from the corresponding author on reasonable request.
